# Artificial Neural Network Modeling of Quality of Life of Cancer Patients: Relationships between Quality of Life Assessments, as Evaluated by Patients, Pharmacists, and Nurses

**Published:** 2011-12

**Authors:** Rieko Takehira, Keiko Murakami, Sirou Katayama, Kenji Nishizawa, Shigeo Yamamura

**Affiliations:** 1 *Faculty of Pharmaceutical Sciences, Josai International University, Gumyo 1, Togane, Chiba, Japan;*; 2 *Department of Pharmacy, Nippon Medical University Hospital, Sendagi 1-1-5, Bunkyo-ku, Tokyo, Japan;*; 3 *Department of Pharmacy, Omori Medical Center, Toho University, 6-11-1 Omori-Nishi, Ota-ku, Tokyo, Japan*

**Keywords:** quality of life, neural network, pharmacist, nurse, profession

## Abstract

**Aim:**

The purpose of this study was to investigate the difference between the professional perspectives of pharmacists and nurses in Japan with regard to evaluation of the quality of life (QOL) of cancer patients.

**Methods:**

A group of cancer hospital inpatients (n=15) were asked to rate the condition of their health and their QOL by filling in a questionnaire. On the same day, a group of pharmacists (n=8) and nurses (n=18) also evaluated patient QOL. Three-layered artificial neural network (ANN) architecture was used to model the relationship between the different QOL evaluations made by patients, pharmacists, and nurses.

**Results:**

Although there was no statistical difference between the QOL scores obtained from pharmacists and nurses, the correlation between these scores was weak (0.1188). These results suggest that pharmacists and nurses evaluate the QOL of their patients from different perspectives, based on their respective profession. QOL parameters were modeled with an ANN using the scores, given by patients in answer to questions regarding health-related QOL as input variables. Both the predictive performance of the ANN and the robustness of the optimized model were acceptable. The response surfaces calculated by ANN modeling showed that pharmacists and nurses evaluate patient’s QOL using different information and reasoning, which is likely related to the nature of their contact with the patients.

**Conclusion:**

Health professionals evaluate patient QOL from different perspectives, depending on their profession.

## INTRODUCTION

Cancer patients tend to experience increasing pain as disease stage progresses. Opioid preparations are used to relieve cancer pain, according to the three-step analgesic ladder established by the World Health Organization (1). Relief from pain, together with the management of adverse events, with analgesics results in an improved quality of life (QOL) for cancer patients, which is the most important issue for medical professionals such as doctors, pharmacists, and nurses (2).

The health-related quality of life (HRQOL) of patients is a concept that consists of a variety of elements, including emotional well-being (EWB), functional well-being (FWB), social well-being (SWB) and physical well-being (PWB) (3). When providing patient care it is important to take facilitation of an improvement in their HRQOL into account. HRQOL, as rated by patients, is a subjective element, and objective evaluations undertaken by pharmacists and nurses may differ, due to the differences in their professional roles in patient care (4).

We have previously established a structural equation model (SEM) of subjective QOL scores in cancer patients in two publications, in which we accounted for the fact that pharmacists possess the ability to improve these scores (5, 6). In the first investigation, the SEM-modeled QOL was poorly correlated with subjective patient assessment of QOL and the competency of pharmacists was found to improve subjective patient’s QOL. In the second study, we revealed that the relationship between patient assessment of QOL and the competency of pharmacists was robust for missing values. Artificial neural networks (ANNs) are powerful tools to use in the simulation of various nonlinear systems, and they have been applied to both risk evaluation and prediction of prognosis in medical science (7, 8). We have previously applied ANN to predict the pharmacokinetics/pharmacodynamics of antibiotics in patients with severe burns (9-11). In the present study, we applied ANN modeling to predict the QOL of cancer patients as perceived by them and evaluated by pharmacists and nurses using the same parameters used in the SEM model established in the previous paper (5). The aim was to evaluate the relationship between subjective patient QOL assessments and QOL assessments made by pharmacists and nurses.

## METHODS

### Participants

Patients: A group of cancer patients (n=18) hospitalized in Nippon Medical University Hospital (Sendagi, Tokyo Japan) were initially included in this study. All patients took opioid analgesics for pain control, and a pain control team, organized by physicians, pharmacists and nurses, provided appropriate in-hospital care. Patients were excluded if they began chemotherapy during the study period, or if they did not complete the questionnaire, owing to the severity of their illness. Thus, 15 patients (eight females and seven males, age: 64.7 ± 7.2 yrs: mean ± SD) were enrolled in the study and gave written consent to answer the study questions. A questionnaire was designed to assess the HRQOL of patients referring SF36 (12), Functional Living Index-Cancer (FLIC) (13), and Functional Assessment of Cancer Therapy: General (FACT-G) (14); it consisted of four important domains: EWB, FWB, SWB, and PWB (3). The number of questions included was limited to 18 in order to avoid unnecessary burden on the patients, in accordance with the suggestion of a local research committee. Patient health-related status and subjective QOL were collected by pharmacists in the form of a bedside interview and data collection was conducted four times every week, using a questionnaire (5). Time required to fill the questionnaire by interviewing was about 5-10 minutes.

Pharmacists and nurses: Pharmacists (n=8) and nurses (n=18) providing patient care in a pain control team were involved in this study. Details regarding the amount of professional experience possessed by the participating pharmacists and nurses are listed on Table 1. Pharmacists evaluated patient QOL when interviewing patients using the questionnaire. Nurses evaluated patient QOL on the same day as the patient answered the questionnaire. Patient QOL was evaluated on a simple scale ranging from 1 (very bad) to 5 (very good), rather than in a structured manner. The intended number of the answers in the research was 60 (each of 15 patients would answer 4 times). However, some patients, pharmacists, and nurses did not complete the questionnaires, so a number of paired (patient, pharmacist, and nurse) forms (n=40) were used in the analysis. Table 2 shows the items of the questionnaires which were selected to be used for the SEM (5) and mean values of their score, as well as the mean QOL scores given by patients, pharmacists, and nurses.

**Table 1:** Background status of participating pharmacists (n=8) and nurses (n=18)

	<1 year	1 to 5 years	>5 years

Years of experience working as pharmacists	2	0	6
Years of experience working in a pain control team	2	5	1
Years of experience working as a nurse	0	13	5
Years of experience working in a pain control team	4	11	3

**Table 2:** Questions and Scores of items (mean ± S.D.) answered by patients, pharmacists and nurses

Item	Questions	Mean ± S.D.	

Q1	Did you sleep well?	4.10 ± 1.03	
Q2	Do you worry about your pain and/or nausea?	2.50 ± 1.34	
Q3	Have you felt unable to concentrate?	3.10 ± 1.24	
Q4	Did you experience nausea?	2.33 ± 1.59	
Q5	Did you vomit?	1.63 ± 1.17	
Q6	Rate of your pain	3.18 ± 1.74	
Q7	Did you enjoy a book or radio or television program?	2.58 ± 1.36	
Q8	Were you able to move freely to a rest room without assistance?	2.70 ± 1.91	
QOL (Patients)	Self-evaluated by patients	2.50 ± 0.82	*p*=0.0003^a^
QOL (Pharmacists)	QOL of patients evaluated by pharmacists	3.05 ± 0.93	*p*=0.0025^a^
QOL (Nurses)	QOL of patients evaluated by nurses	3.13 ± 0.94	*p*=0.7649^b^

Q1–Q3, EWB; Q4–Q6, PWB; Q7–Q8, FWB. Answers were scores ranging from 1 to 5 except for Q6. Answers to Q6 were rated on a scale ranging from 0 to 10.

a *p* values calculated by Wilcoxon signed-rank test of difference between QOL evaluated by patients and health providers;

b *p* value calculated by Wilcoxon signed-rank test of difference between QOL evaluated by pharmacists and nurses.

The study design and questionnaires were reviewed by a local research committee. The background of the patients and details of the questionnaires they were given are described in our previous study (5).

### ANN

A three-layered ANN architecture was used and optimization of the weights between neurons to match the evaluated QOLs with those that were predicted was carried out using a second-order, conjugate, gradient descent algorithm (15). In this algorithm, a search is performed along conjugated directions, which generally produce faster convergence compared with a back-propagation of the error algorithm (15).

Scores obtained from patients are shown in Table 2 and were used for input data (independent parameters). These 8 questions were from the initial 18 questions and sufficed to perform exploratory factor analysis (5). The subjective patient QOL scores and QOL evaluations made by pharmacists and nurses were used for output data (dependent parameters). The determination of the number of neurons in the hidden layer will be described subsequently. The optimized ANN model had initial value dependence, so at least 10 runs were performed using reinitialized weights between neurons, after which the model with the best fit between observations and predictions from the training data was adopted as the optimized ANN model.

Statistica 06J, featuring a neural networks module (StatSoft Japan, Tokyo, Japan), was used for ANN calculation. A sigmoid function was adopted for activation function of the hidden layer. Robustness of optimized ANN was investigated with leave-one-out cross-validation. The procedure is as follows: The data obtained from one patient was removed from the data set and data from the remaining patient were used as the training data set. The ANN was optimized using the training data set, then the outcome of the excluded patient was predicted by the optimized ANN model.

### Statistical analysis

Other statistical analyses were carried out with SPSS 18.0J, AMOS 18J (SPSS Japan, Tokyo, Japan) and JMP 8J (SAS institute Japan, Tokyo, Japan).

## RESULTS AND DISCUSSION

### QOL evaluated by patients, pharmacists and nurses

As shown in Table 2, the subjective QOL scores given by patients were significantly lower than those given by both pharmacists and nurses, and the latter did not show statistical difference (*p*=0.7649 by Wilcoxon signed-rank test). At least to compare among QOL scores given by patients, pharmacists and nueses, pharmacists and nurses may have a tendency to underestimate the condition of the patients. Table 3 shows the Spearman’s correlation coefficient between the QOL scores given by patients, pharmacists, and nurses. The correlation between patient and pharmacist scores was moderate (*r*=0.4481), and the correlation between the scores of patients and nurses was very weak to negligible (*r*=0.1187). The correlation between those QOL scores given by pharmacists and those given by nurses were also very weak to negligible (*r*=0.1188).

**Table 3:** Spearman’s correlation coefficients of QOL scores evaluated by patients, pharmacists, and nurses

	QOL evaluated by patients	QOL evaluated by pharmacists	QOL evaluated by nurses

QOL evaluated by patients	1.0000		
QOL evaluated by pharmacists	0.4481	1.0000	
QOL evaluated by nurses	0.1187	0.1188	1.0000

It has been suggested that doctors would underestimate the number of symptoms experienced by cancer patients (16). However, Sneeuw *et al*., reported that healthcare providers tend to assess patients as having more symptoms than did the patients themselves (17). Some other studies have reported that healthcare providers are likely to underestimate the physical symptoms of patients (18, 19). Our results show that pharmacists and nurses seem to have the same tendency as doctors to underestimate the condition of health of patients. Furthermore, although there were no statistically differences in QOL as evaluated by pharmacists and nurses (*p*=0.7649), the correlation between them was very weak to negligible (*r*=0.1188). These results suggest that pharmacists and nurses evaluate the QOL of their patients from different perspectives, based on their respective profession.

### ANN model for QOL of patients

**Figure 1:**
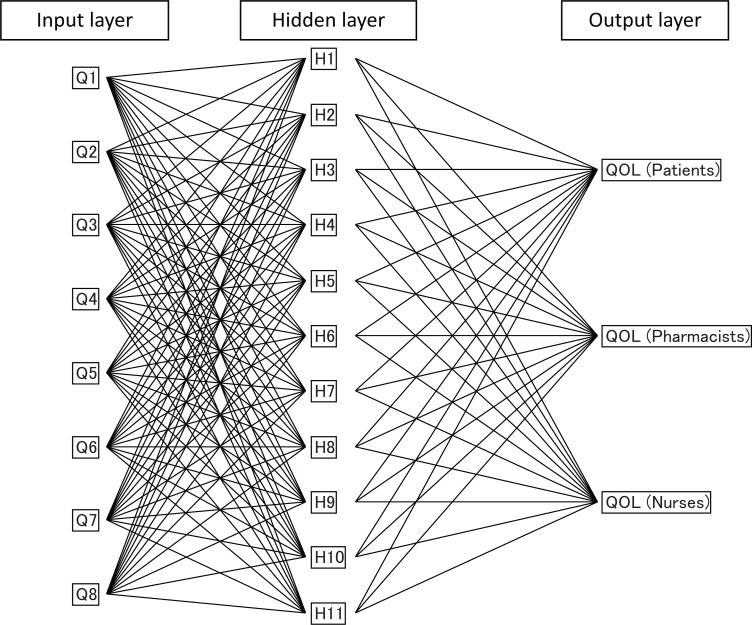
ANN diagram to model the QOL of cancer patients.

We have previously reported that the QOL of cancer patients was modeled well with a score of eight answers (Table 2) in the questionnaire, using SEM (5). As described, pharmacists and nurses evaluate the QOL of their patients from different professional perspectives. We used an ANN to investigate the difference in perspectives between pharmacists and nurses with regard to evaluation of QOL using. As ANN architecture, we used a three-layer perceptron: an input layer comprised of eight processing elements (the scores obtained from the answers to the questions), a hidden layer, comprised of processing elements with a sigmoid function as an activation function, and an output layer comprised of the QOL scores obtained from patients, pharmacists, and nurses. The network diagram that was used in the present investigation is shown in Figure 1. The neurons in the hidden and output layers work to calculate the sum of products of values of previous layers and the weight between connections. The neurons then transfer a value to neurons in the next layer according to an activation function (20). All weights among neurons were optimized to minimize differences between observed and modeled QOLs.

**Figure 2:**
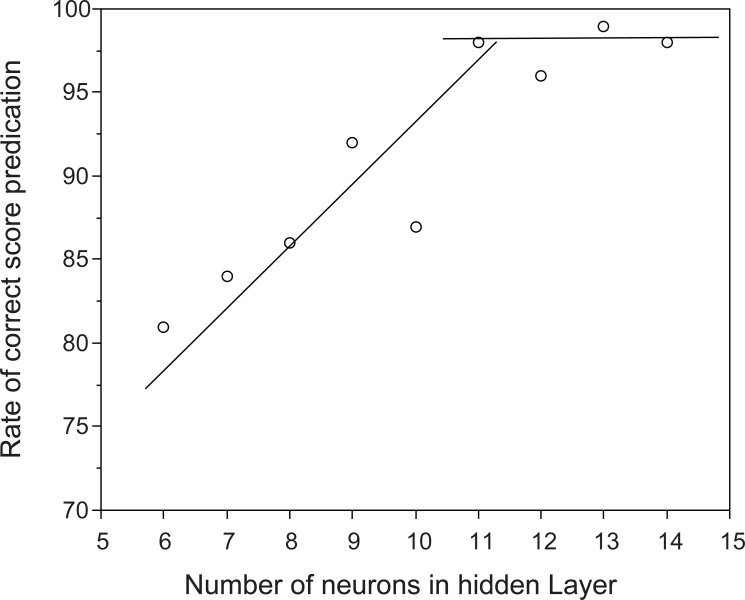
Change of prediction performance of QOL with number of neurons in hidden layer.

Figure 2 shows the effect on prediction performance of QOL of the number of neurons in the hidden layer, using the ANN model. The best fit was obtained when more than 11 neurons were arranged in the hidden layer. In order to avoid “over-fitting” a smaller number of neurons is preferable, so a three-layered architecture with 11 neurons in the hidden layer was used for modeling in this study (Fig. 1) (20).

Table 4 shows the prediction performance of QOL as evaluated by patients, pharmacists, and nurses using the ANN model. In the final model, subjective QOL, as assessed by patients, and the QOL scores given by pharmacists were all successfully predicted, and only a few of the data obtained from nurses were not predicted by the ANN model that was established. These results suggest that the necessary information to predict how pharmacists would evaluate QOL is contained in the input data.

**Table 4:** Prediction performance of QOL by ANN modeling

QOL score	QOL evaluated by patients		QOL by evaluated pharmacists		QOL evaluated by nurses	
	Answered	Predicted^1)^	Answered	Predicted^1)^	Answered	Predicted^a^

5	0	0	1	1	2	1
4	5	5	15	15	11	11
3	13	13	9	9	20	19
2	19	19	15	15	4	4
1	3	3	0	0	3	3
Performance^b^	100.0		100.0		95.0	

a Number of correct scores predicted;

b Performance is the rate of correct scores predicted (%).

The robustness of the ANN model was evaluated with the leave-one-out cross-validation. Table 5 shows the prediction performance of QOL with leave-one-out cross-validation. The rate of correct prediction was approximately 60% for the QOL scores obtained from patients, pharmacists, and nurses, which seems to indicate that the use of the ANN model to predict QOL is not robust. However, only 2/40 patients, 5/40 pharmacists, and 6/40 nurses had differences between evaluated and predicted QOL that were greater than 1 (results not shown). These results indicate that approximately 90% of QOL data (from 107/120 individuals) could only be roughly, rather than precisely, predicted by the ANN model. QOL is a broad concept, including not only the condition of physical health, but also mental health, education, and social belonging (21). The patients evaluated their QOL subjectively, based not only on the condition of their own health, but also on their concept of values (22). We argue that pharmacists and nurses scored patients QOL primarily based on the condition of health of each patient, as assessed from their professional perspective. Therefore, it would be very difficult to make a precise prediction of patient QOL score using data from health professionals. Furthermore, each respective patient was not evaluated by a particular pharmacist and nurse every time. This may have lead to individual differences in the evaluation of QOL. If these were considered, a roughly predictive performance of approximately 90% by ANN would be acceptable.

**Table 5:** Robustness of optimized ANN evaluated by leave-one-out cross-validation

	QOL evaluated by patients		QOL evaluated by pharmacists		QOL evaluated by nurses	
	Answered	Predicted^1)^	Answered	Predicted^1)^	Answered	Predicted^a^

5	0	0	1	0	2	0
4	5	4	15	8	11	8
3	13	8	9	3	20	12
2	19	13	15	13	4	1
1	3	1	0	0	3	1
Performance^b^	65.0		60.0		55.0	

a Number of correct scores predicted;

b Performance is the rate of correct scores predicted (%).

### Perspective of pharmacists and nurses for evaluation of patient QOL

**Figure 3:**
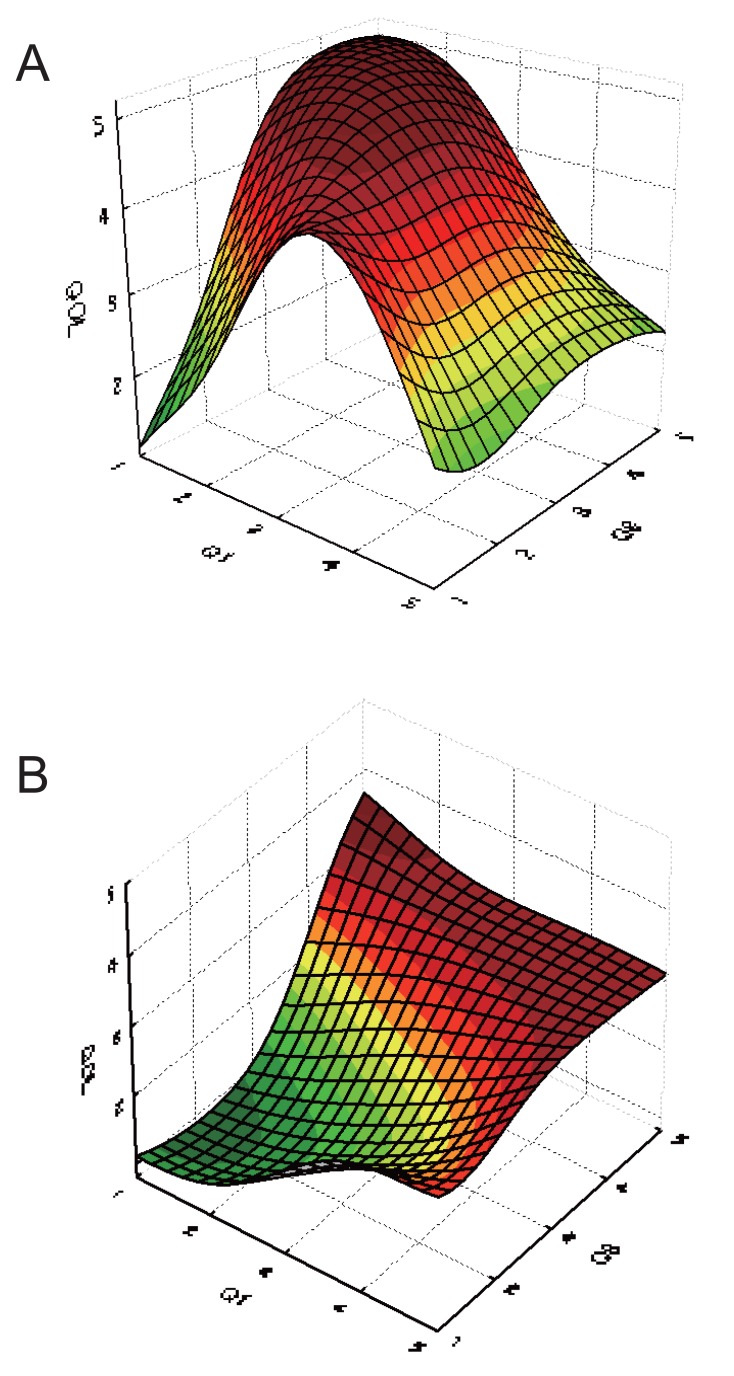
Response surface of input parameters and QOLs obtained from ANN model. Q1 (Did you sleep well?)-Q8 (Were you able to move freely to a rest room without assistance?)-QOL. (A), evaluated by pharmacists; (B), evaluated by nurses.

**Figure 4:**
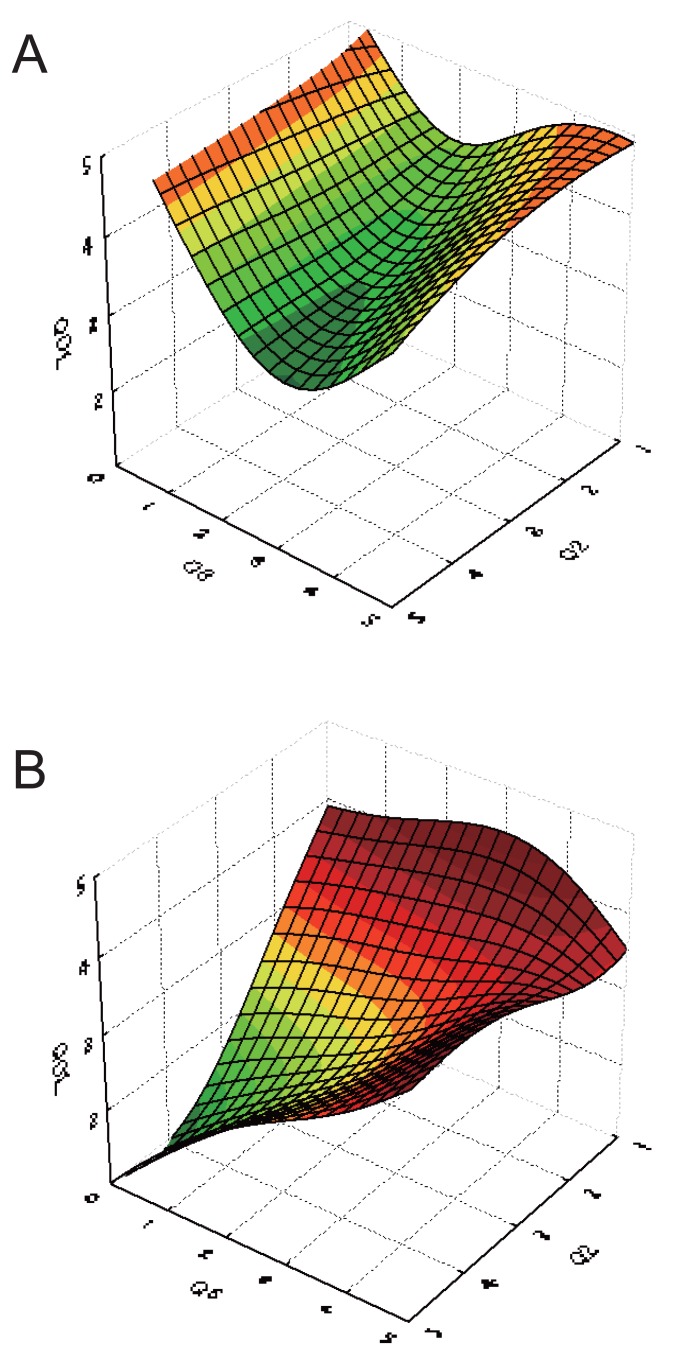
Response surface of input parameters and QOLs obtained from ANN model. Q2 (Do you worry about your pain and/or nausea?)-Q6 (Rate of your pain)-QOL. (a), evaluated by pharmacists; (b), evaluated by nurses.

The difference in perspectives between pharmacists and nurses in evaluation of patients’ QOL was investigated by visualization of plots of response surfaces of the exploratory variables and one response variable of the optimized ANN model used. While many of the response surfaces of relationships between two sets of input data and QOL scores assessed by pharmacists and nurses are similar to each other, some of the response surfaces were different, indicating a difference in perspectives between pharmacists and nurses. Figures 3 and 4 show the response surfaces between two sets of input data and QOL as evaluated by pharmacists and nurses, indicating a strong difference between pharmacists and nurses.

Figure 3 shows the response surfaces indicating the relationship between “Q1 (Did you sleep well?),” “Q8 (Were you able to move freely to a rest room without assistance?),” and QOL as evaluated by pharmacists and nurses. The response surface should indicate that QOL score was higher when the scores associated with the answers to Q1 and Q8 were higher, i.e. “good sleep” and “good mobility.” Pharmacists evaluated QOL as lower when patients answered “good sleep” (a higher score in answer to Q1), and would also evaluate QOL as lower if patients answered that they had a good sleep after taking a sleeping medication. This may be explained since pharmacists may reason that if patients need to take such medication, then the condition of their health must be poor. The response surface of QOL as evaluated by nurses indicated that nurses regarded QOL as higher when patients answered “good sleep” and “good mobility.” Furthermore, nurses evaluated patient QOL with greater sensitivity towards mobility than towards sleep, suggesting that nurses would considered that having a good mobility is more important than to sleep well.

Figure 4 shows the relationship between “Q2 (Are you worried about your pain and/or nausea?),” “Q6 (Rate your pain),” and QOL as assessed by pharmacists and nurses. The response surface should indicate that QOL would be higher when scores of Q2 and Q6 were lower (less worry and less pain, respectively). The change of QOL evaluation by pharmacists with Q2 and Q6 was small compared to that evaluated by nurses (Fig. 4b).

The response surface for QOL evaluated by nurses indicated that when less worry was felt (indicated by a lower score in Q2), nurses regarded QOL as higher, independent of pain (the score given in answer to Q6). And when patients were very worried by their condition (higher score in answer to Q2), QOL was related to pain score. The response curve in nurses was hard to explain reasonably. The relation between variables seems complicated and other variable may affect the response curve.

The QOL evaluated by pharmacists are related to sleep and mobility, and less worry. Pharmacists may take into account sleeping medication on QOL evaluation of patients. On the otherhand, QOL evaluated by nurses are related to sleep, mobility, worry and pain. However, effect of the pain on QOL was hard to explain. There would be any confounding factors between the pain and QOL. There are some limitations of this study: 1) Since this study was carried out in Japan, different model would be optimized if the same research were carried out in other countries. 2) Because the number of sample size for analysis, especially the number of pharmacists, is not enough, a statistical powder to obtain the robust model would not be fully satisfactory.

## CONCLUSION

The QOL of cancer patients was evaluated by the patients themselves, and by pharmacists, and nurses on same day. When QOL was self-evaluated by the patients, the scores were different from the QOL scores obtained from pharmacists and nurses. The correlation between QOL scores given by patients and those given by pharmacists and nurses was low. Although the QOL scores given by pharmacists and nurses were not different statistically, the correlation coefficient between them was weak to negligible (*r*=0.1188). These results suggest that pharmacists and nurses evaluate the QOL of their patients from different perspectives, based on their respective profession. The QOL scores were modeled using the scores regarding the HRQOL of patients as input variables using an ANN with three-layer architecture. The predictive performance given by ANN and the robustness of the model were acceptable. The response surface obtained from the optimized ANN model showed the difference in perspectives between the pharmacists and nurses in their evaluations of QOL. Health professionals affect QOL scores as a result of the difference of the profession-based perspectives they hold.
